# Reply to “AI-assisted peer review: enhancing evaluation while increasing author workload”

**DOI:** 10.1093/haschl/qxag159

**Published:** 2026-06-27

**Authors:** Howard Bauchner

**Affiliations:** Boston University Chobanian & Avedisian School of Medicine, Boston, MA, United States; National University of Singapore, Singapore

**Keywords:** AI, peer-review, AI peer-review, LLM

Dr Mondal^1^ makes 2 comments worthy of reflection. First, what appears to be undisclosed use of artificial intelligence (AI) by peer-reviewers. I suspect this is becoming more common, despite instructions from many journals not to use AI. Peer-reviewers are overworked, and usually not compensated, so it is not surprising that they are engaging with large language models (LLMs) as an aid. And certainly, if journals begin to use AI it would be inconsistent for them to instruct peer-reviewers not to use it. The answer—transparency. Journals should relax their instructions to authors, which many have, and peer-reviewers and simply request that they acknowledge use of AI. This is much like the early use of preprints; many editors were concerned, but it has become acceptable. Second, the insufferable long reviews by AI. Recently, a colleague shared with me several reviews—one must have been over 3000 words, almost the length of the manuscript. In addition, my own experience suggests that this is a problem! It is why Dr Rivara and I believe that AI must be a companion for editors. Ultimately, if a revision is requested, editors need to direct authors to those comments that they need to respond to—for both AI and peer-reviewers. I do believe, over time, AI will increasingly focus on important issues, rather than just a litany of suggestions, as well as adherence to the appropriate reporting guideline. We are in the early learning phase and beginning to gather experience—better times are ahead.

## Supplementary Material

qxag159_Supplementary_Data

## Data Availability

They are fine to use.
